# Clinical, MRI, and histopathological findings of congenital focal diplomyelia at the level of L4 in a female crossbred calf

**DOI:** 10.1186/s12917-020-02580-4

**Published:** 2020-10-21

**Authors:** Gerlinde J. Wunderink, Ursula E. A. Bergwerff, Victoria R. Vos, Mark W. Delany, Dorien S. Willems, Peter R. Hut

**Affiliations:** 1 Department of Public Health Sciences, Division of Farm Animal Health, Faculty of Veterinary Medicine, Utrecht University, Utrecht, The Netherlands; 2 Division of Pathology, Faculty of Veterinary Medicine, Utrecht University, Utrecht, The Netherlands; 3 Department of Clinical Sciences, Division of Diagnostic Imaging, Faculty of Veterinary Medicine, Utrecht University, Utrecht, The Netherlands

**Keywords:** Myelodysplasia, Congenital diplomyelia, Bunny-hop, Schmallenberg virus, Calf, Ataxia & Pollakiuria

## Abstract

**Background:**

This case report describes the clinical signs of a calf with focal diplomyelia at the level of the fourth lumbar vertebra. Magnetic resonance imaging (MRI) images and histological findings of the affected spinal cord are included in this case report. This case differs from previously reported cases in terms of localization and minimal extent of the congenital anomaly, clinical symptoms and findings during further examinations.

**Case presentation:**

The calf was presented to the Farm Animal Health clinic, Faculty of Veterinary Medicine, Utrecht University, with an abnormal, stiff, ‘bunny-hop’ gait of the pelvic limbs. Prominent clinical findings included general proprioceptive ataxia with paraparesis, pathological spinal reflexes of the pelvic limbs and pollakiuria. MRI revealed a focal dilated central canal, and mid-sagittal T2 hyperintense band in the dorsal part of the spinal cord at the level of the third to fourth lumbar vertebra. By means of histology, the calf was diagnosed with focal diplomyelia at the level of the fourth lumbar vertebra, a rare congenital malformation of the spinal cord. The calf tested positive for Schmallenberg virus antibodies, however this is not considered to be part of the pathogenesis of the diplomyelia.

**Conclusions:**

This case report adds value to future clinical practice, as it provides a clear description of focal diplomyelia as a previously unreported lesion and details its diagnosis using advanced imaging and histology. This type of lesion should be included in the differential diagnoses when a calf is presented with a general proprioceptive ataxia of the hind limbs. In particular, a ‘bunny-hop’ gait of the pelvic limbs is thought to be a specific clinical symptom of diplomyelia. This case report is of clinical and scientific importance as it demonstrates the possibility of a focal microscopic diplomyelia, which would not be evident by gross examination alone, as a cause of hind-limb ataxia. The aetiology of diplomyelia in calves remains unclear.

**Supplementary information:**

**Supplementary information** accompanies this paper at 10.1186/s12917-020-02580-4.

## Background

Diplomyelia is a specific type of myelodysplasia which is defined as an isolated, true duplication of the spinal cord within one set of meninges, and is an important differential in spinally located neurological signs in calves. This case report aims to generate a better understanding of the clinical signs of focal diplomyelia in a calf, in addition to providing magnetic resonance imaging (MRI) images and histological findings of the affected spinal cord. This case differs from previously reported cases of diplomyelia, such as described by Hut et al. [1] and Testoni et al. [2], in terms of localization and extent of the congenital anomaly, clinical signs and findings during further examinations. In addition, to the authors’ knowledge, this is the first time a focal diplomyelia has been reported in a calf. This case report is of relevance to clinical practice due to the fact that diplomyelia should be on the list of differential diagnoses when a calf is presented with ‘bunny-hop’-locomotion since the calf’s birth. In particular it demonstrates the importance of MRI and microscopic examination when gross lesions are not apparent.

## Case presentation

### History

At 2 weeks of age, a female cross-bred (62.5% Holstein Friesian, 25% Jersey, 12.5% Brown Swiss) calf was presented to the Farm Animal Health clinic, Faculty of Veterinary Medicine, Utrecht University (Utrecht, The Netherlands). The reason for referral was the presence of ‘bunny-hop’-locomotion since the calf’s birth. According to the farmer, the parturition process of the multiparous dam was rapid and without complications. The calf was active, alert, and suckled quickly after birth. The next day, the calf moved forward by taking regular steps with the thoracic limbs, whereas the pelvic limbs were stiff and moved as a single ‘bunny-hop motion. There was no history of bovine herpes virus-1 (BHV-1), leptospirosis, neosporosis, Johne’s disease or salmonellosis on the dairy farm. During gestation and parturition period of this calf there were f several premature births on the farm, multiple positive Bovine Viral Diarrhea Virus (BVDV) cases, and one confirmed BVDV-carrier calf.

### Clinical examination

**Fig. 1:**
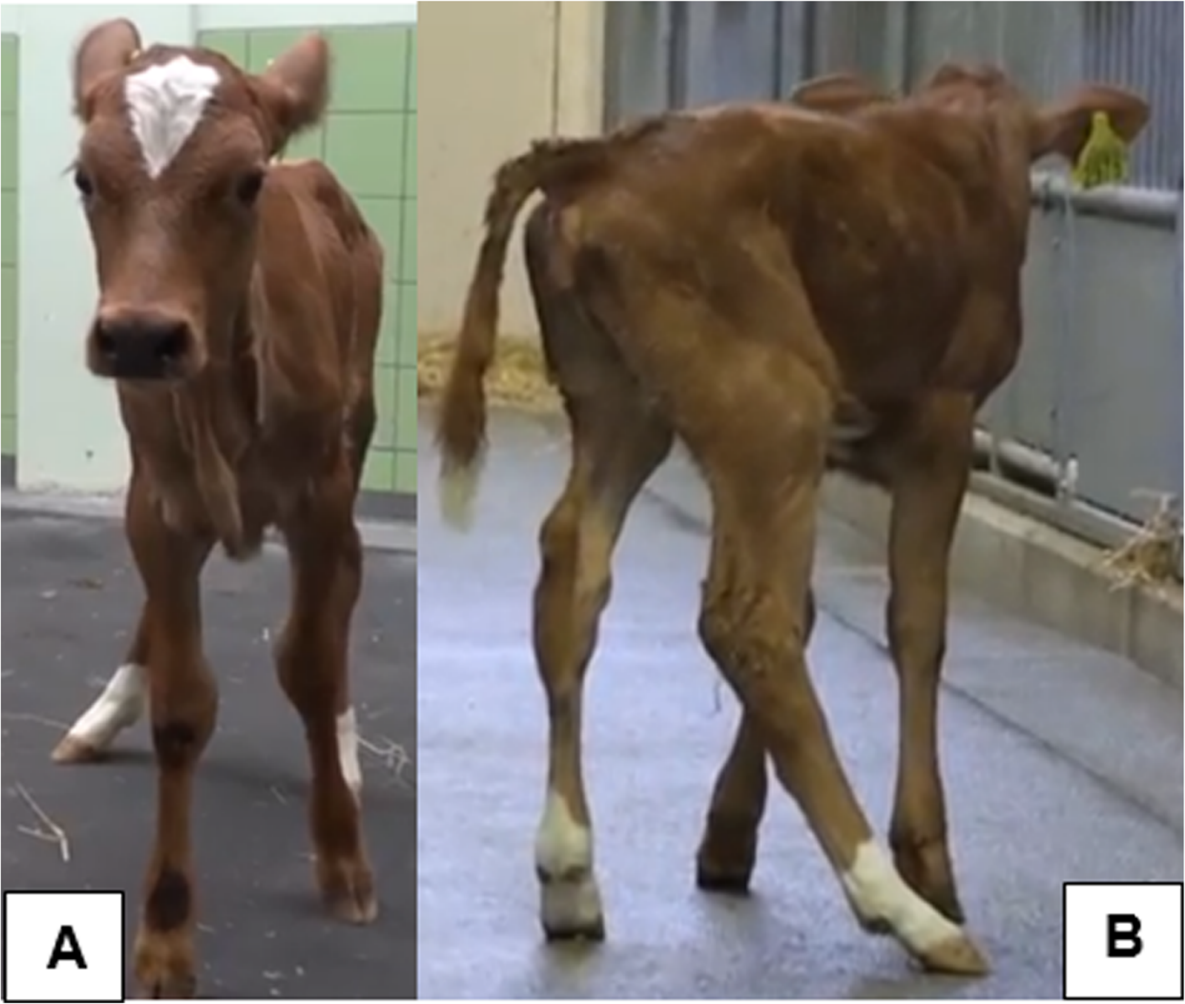
Clinical presentation of the calf at the age of 2 weeks. Clinical presentation of the calf at the age of 2 weeks (**a**: front view & **b**: right hind side view). The pelvic limbs show muscle atrophy, are placed more cranially than normal and the right pelvic limb shows a distal valgus in combination with a reduced extension in the tarsal joint when compared to the left pelvic limb

At 2 weeks of age, the calf was examined at the Farm Animal Health Clinic, Faculty of Veterinary Medicine, Utrecht University (Utrecht, The Netherlands). The calf appeared to be bright and alert, no abnormal behaviour was noticed. It showed normal eating, drinking, and defecation. The animal had a body condition score of 2/5 and bilateral, symmetrical, muscle atrophy of the pelvic limbs. Dysuria was present as pulsatile dribble of small jets of urine which occurred intermittently. In between these periods of active urination passive leakage of small amounts of urine was observed. The calf was able to stand on its own, however during clinical examination a slow increase in bending of the legs was noticed and eventually the calf became recumbent l. The posture of the spine of the calf appeared to be slightly kyphotic. Both thoracic limbs showed no abnormalities in position, whereas the pelvic limbs appeared to be hypertonic with marked bilateral, symmetrical, muscle atrophy. Seemingly due to a lack of proprioception, the calf placed its pelvic limbs more cranially than normal, in addition the left pelvic limb was more extended in the tarsal joint than the right pelvic limb, and the right pelvic limb showed a distal valgus (Fig. 1).

While walking and running the calf showed a gait abnormality; normal movement of the thoracic limbs, abnormal ‘bunny-hop’ gait of the pelvic limbs, general proprioceptive ataxia affecting the pelvic limbs, and both pelvic limbs drifted laterally towards the left. No abnormalities were observed on clinical examination of other organ systems.

Neurological examination [3] showed no abnormal functioning of the cranial nerves. Pupillary light reflex, palpebral reflex and corneal reflex were all within normal limits. The menace response was absent, but this was most likely age-related [4]. Postural reactions were present and rapid in the thoracic limbs, and absent in the pelvic limbs. The bilateral thoracic spinal reflexes, and patellar reflexes were within normal limits. However, the withdrawal reflex of either one of the pelvic limbs caused exaggerated flexion of both pelvic limbs simultaneously. Nociception was not tested, because the calf showed a positive withdrawal reflex.

**Fig. 2:**
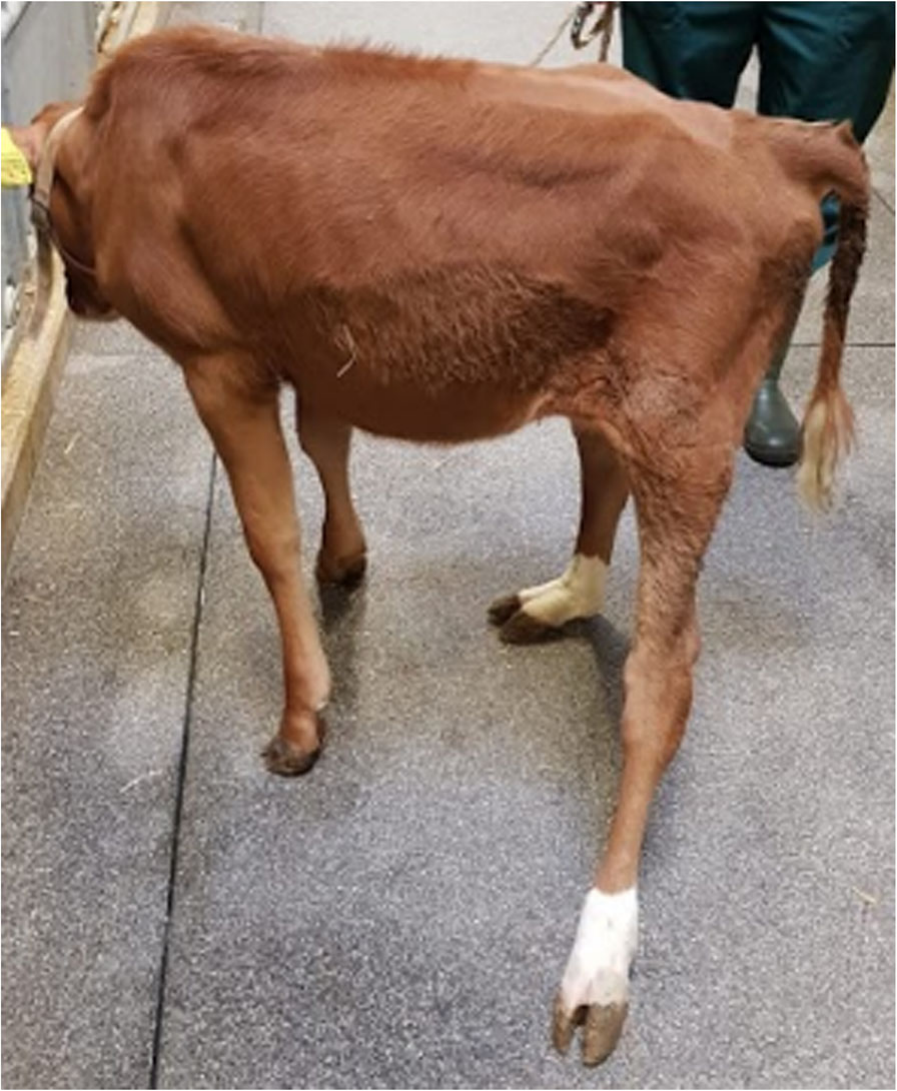
Clinical presentation of the calf at the age of 8 weeks. Clinical presentation of the calf at the age of 8 weeks. The pelvic limbs show muscle atrophy and the left pelvic limb shows a distal valgus in combination with a reduced extension in the tarsal joint when compared to the right pelvic limb

At the age of 8 weeks, a re-evaluation of the clinical symptoms showed some similarities and differences in comparison to the previously described symptoms at 2 weeks of age. Pollakiuria was still present and the pelvic limbs remained hypertonic with marked bilateral, symmetrical, muscle atrophy. However, at the age of 2 weeks the left pelvic limb was more extended in the tarsal joint than the right pelvic limb, whereas at the age of 8 weeks the right pelvic limb was more extended in the tarsal joint than the left pelvic limb. In addition, at the age of 2 weeks the right pelvic limb showed a distal valgus, whereas at the age of 8 weeks the left pelvic limb showed a distal valgus (Fig. 2).

While walking and running, the calf showed a grade 3/5 gait abnormality [3], normal movement of the thoracic limbs, abnormal ‘bunny-hop’ gait of the pelvic limbs, general proprioceptive ataxia affecting the pelvic limbs, and both pelvic limbs drifted laterally towards the right. An additional movie file shows this in more detail (see Additional file 1).

Neurological examination [3] showed no abnormal functioning of the cranial nerves, and when confronted with obstacles (broom) there were no clinical indications of eyesight related problems. Postural reactions were tested, however the calf tended to fall each time a leg was lifted. Therefore the outcome of the proprioceptive tests was difficult to interpret. Spinal reflexes were assessed: the pannicular reflex, patellar reflex, and perineal reflex were present, and showed no abnormalities. The withdrawal reflexes of the thoracic limbs were within normal limits. The withdrawal reflex of the right pelvic limb was delayed and reduced in strength. The withdrawal reflex of the left pelvic limb was completely absent. The muscle tone was examined by placing the calf in lateral recumbency, and passive flexion of the leg was performed. The thoracic limbs showed a normal counterforce when flexing the leg, and all of the joints could be maximally flexed. The pelvic limbs showed a strong increase in counterforce when the leg was flexed on examination. The range of motion of both hips and tarsal joints was reduced to a couple of centimetres.

Repeated neurological examination, at the age of 10 weeks, showed similar results, however some additional neurological abnormalities were evident. The withdrawal reflex was present in both thoracic limbs, whereas it was absent in both pelvic limbs. In addition, the patellar reflex of both pelvic limbs was delayed and reduced in strength.

In summary, the following clinical problem is defined as: a female crossbred calf with congenital ‘bunny-hop’-locomotion, proprioceptive ataxia affecting the pelvic limbs, bilateral muscle atrophy in both pelvic limbs, increased extensor tonus of the pelvic limbs, altered spinal reflexes in the pelvic limbs, and pollakiuria.

An increased extensor tonus in the pelvic limbs indicates a defect in the upper motor neurons, and the fact that the patellar reflex is reduced shows that this defect is located between the fourth and fifth lumbar vertebra (L4-L5) [3]. Bilateral absence of the withdrawal reflex in the pelvic limbs indicates a defect at the level of the fifth lumbar vertebra (L5), sixth lumbar vertebra (L6) or first sacral vertebra (S1, 3). Thus, the neuroanatomical localization of the spinal defect was addressed to the upper motor neurons in the spinal cord between L4 and S1.

### Differential diagnosis

Regarding the main groups of disease categories, certain groups are more likely than others to have caused the symptoms of this calf. For example, the clinical signs of the calf could be explained by a traumatic incident due to assisted delivery or dystocia, however the birth of this calf was described as fast and without problems. Vascular problems, such as ischemic or haemorrhagic infarctions around the time of birth, seem less likely in this calf, as some gradual functional improvement would be expected over time [5, 6]. Metabolic//toxic spinal cord diseases are not recognized in animals, and secondly no idiopathic spinal cord diseases without detectable lesions are known in calves. Degenerative diseases are typically common in later juvenile stages and adulthood, have a slow, insidious onset and a progressive course. This calf showed onset of symptoms at birth without an evident progressive course. Active inflammatory, infectious, degenerative and neoplastic diseases can cause a slow progressive course of the symptoms, but are less likely to cause symptoms from birth onwards. In this case, either congenital anomalies or malformations in the spinal cord between L4 and S1 are most likely to be the cause of the symptoms, although these typically cause a more static progression of the clinical symptoms [1, 2, 7, 8].

Congenital malformations of the spinal cord are called myelodysplasia. Different types of myelodysplasia are among others: hydromyelia, syringomyelia, diastematomyelia, diplomyelia, and dimyelia [9, 10]. Hydromyelia [1] is a condition in which the central canal is absent or dilated. Syringomyelia is a term to describe cavities in the spinal cord, usually located in the white matter. Diastematomyelia is a duplication of the spinal cord with separate vertebral canals and meninges. Diplomyelia [1, 2] is defined as an isolated, true duplication of the spinal cord within one set of meninges. Dimyelia [9] is a generalized duplication of the spinal cord. Segmental hypoplasia is a condition in which some spinal cord segments are smaller than others. Additional investigation was needed to discriminate between the different possible types of myelodysplasia that may have caused the clinical symptoms of this calf.

### Investigations

#### Serology

Serology was indicated as in-utero infection with certain pathogens can possibly lead to congenital malformations in the central nervous system of calves. Therefore, serology performed at the Animal Health Service (Royal GD, Deventer, The Netherlands) for BVD virus antigen and antibodies, Schmallenberg virus antibodies, Bluetongue virus antibodies and *Neospora* antibodies. The test for Schmallenberg virus antibodies was positive.

#### Magnetic resonance imaging (MRI)

**Fig. 3:**
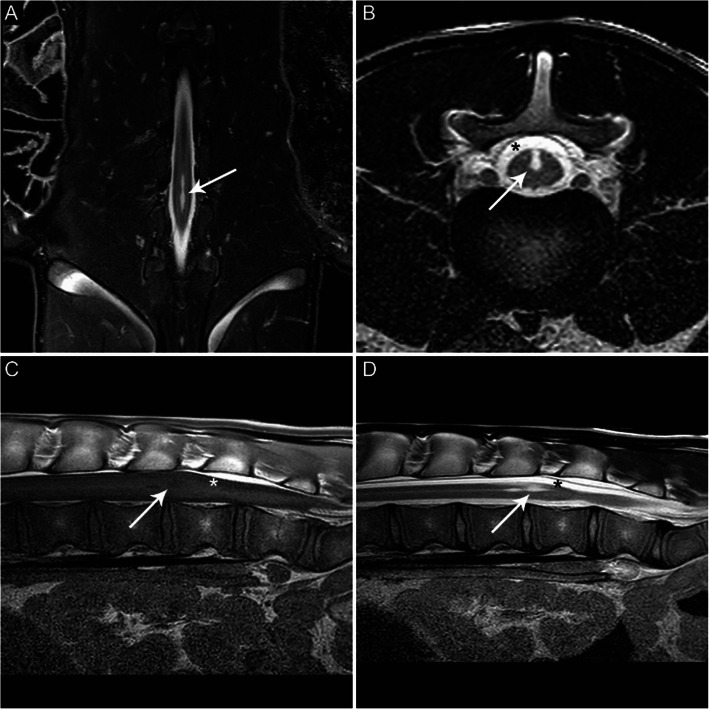
Dorsal STIR (**a**), transverse T2W (**b**), sagittal T1W (**c**), and sagittal T2W (**d**) MRI images. Dorsal STIR (**a**), transverse T2W (**b**), sagittal T1W (**c**), and sagittal T2W (**d**) MRI images. The figure letters denote the cranial and right side in **a**, the dorsal and right side in **b**, and the cranial side in **c** and **d**. The images show a focal well-defined T2 hyperintense widening of the central canal (arrows) in the spinal cord at the level of mid L3 until mid L4, with dorsal extension up to the level of the subarachnoid space (asterisks)

Based on ethical grounds and poor prognosis, the 10-week-old calf was humanely euthanized by means of an intravenous injection containing Euthasol® 500 mg/ml solution conform the instructions on the information leaflet. Directly after euthanasia, an ex vivo Magnetic Resonance Imaging (MRI) scan of the lumbar spinal cord was performed using a 1.5 Tesla scanner (Phillips Ingenia: Phillips Medical Systems Nederland B.V.,Best, The Netherlands) (Fig. 3). MRI was carried out in order to identify the exact location of the spinal anomaly and to determine the type of myelodysplasia that may have caused the clinical symptoms of this calf. The following sequences were acquired: sagittal T1-weighted (T1W) turbo spin echo (TR 547 ms, TE 8 ms, 2.5 mm slice thickness), sagittal T2-weigthed (T2W) turbo spin echo (TR 3321 ms, TE 110 ms, 2.5 mm slice thickness), transverse T2W turbo spin echo (TR 3705 ms, TE 120 ms, 3 mm slice thickness), and coronal short tau inversion recovery (STIR; TR 3840 ms, TE 70 ms, 3 mm slice thickness). The MRI scan showed a mild focal widening of the central canal at the level of the midbody of L3 up to the level of the midbody of L4. A dorsoventrally oriented, well-defined, mid-sagittal T2 hyperintense, T1 hypointense band was noted in the dorsal part of the spinal cord at this level, confluent with the dorsal subarachnoid space and the central canal. This band caused a symmetric splitting of the dorsal part of the spinal cord. A focal, symmetric ±25% thinning of the spinal cord was seen at the level of the L4 caudal endplate. The MRI findings were considered compatible with congenital partial split cord or syringohydromyelia of the lumbar spinal cord (L3-L4).

#### Necropsy

Necropsy and histology were performed at the Veterinary Pathology Diagnostic Centre, Faculty of Veterinary Medicine, Utrecht University, Utrecht, The Netherlands.

#### Macroscopic examination

Necropsy was performed in order to specify the structure, position and type of the spinal anomaly. In addition, the amount of cerebrospinal fluid was assessed and the calf was checked for possible additional abnormalities of the internal organs. Macroscopically the spinal cord was intact with no gross changes identified in its structure, position or surrounding tissues. The spinal canal showed no narrowed areas or other vertebral malformations. A slightly increased amount of cerebrospinal fluid was noted upon excision of the caudal lumbar spinal cord. Further examination of the internal organs revealed no gross abnormalities.

#### Microscopic examination

**Fig. 4:**
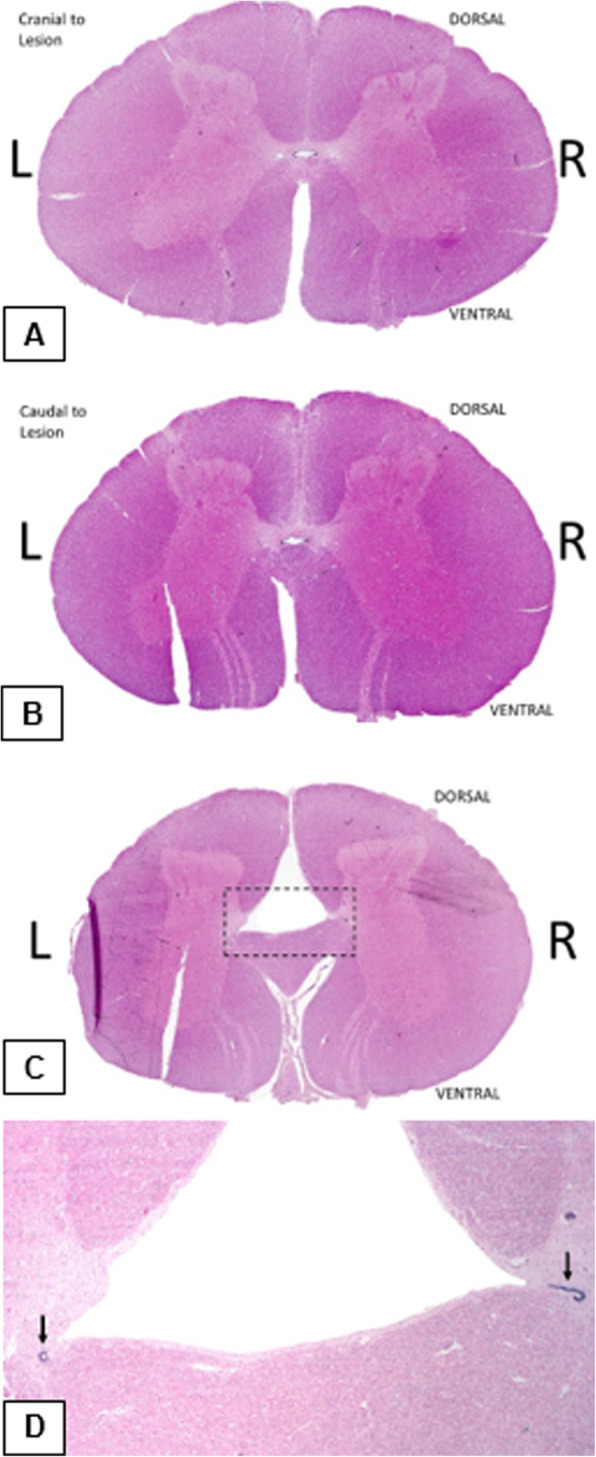
Subgross overview 5 mm cranial from the congenital anomaly (**a**), section 5 mm caudal from the congenital anomaly (**b**), section of the anomaly (**c**), and a 20x view of the anomaly (**d**). Subgross overview 5 mm cranial from the congenital anomaly (**a**), section 5 mm caudal from the congenital anomaly (**b**), section of the anomaly (**c**), and a 20x view of the anomaly (**d**). Image **a** and **b** show a dilatated central canal cranial and caudal to the anomaly associated with a slight elongation of the decussating grey matter. The dashed box in image **c** shows the borders of the magnification, which is shown in image **d**. Image **c** and **d** show epithelial lining of the central canal as a discontinuous layer in the ventrolateral aspects of the dorsal medial sulcus (arrows), an elongated midline bridge of white matter and an absence of grey matter extending across the midline

Histology on the spinal cord at L4 was performed in order to determine the histological structure of the anomality. In addition, histology of the cerebellum was performed to check for possible abnormalities in the cerebellar motor cortex. The spinal cord at L4 showed an increase in the size of the ventral median fissure and the dorsal medial sulcus (Fig. 4c). The epithelial lining of the central canal was presented as a discontinuous layer in the ventrolateral aspects of the dorsal medial sulcus, which did not extend across the midline (Fig. 4d). In addition, the grey matter did not cross the midline. The two sides of the spinal cord were connected by a narrow bridge of white matter which was located ventral to the central canal epithelium (Fig. 4d). Sections, approximately 5 mm cranial and caudal to the above described section, showed a single slightly dilated central canal with an elongation of the decussating grey matter (Fig. 4a and b). Histology of the cerebellum did not show any abnormalities.

## Discussion and conclusions

### Neurological symptoms

The calf displayed ‘bunny hopping’, which has been described as a typical clinical symptom of diplomyelia [11]. It is assumed that bunny hopping is the result of an inability of the interneural communication to coordinate the gait [11]. The lumbosacral area contains inhibitory interneural connections between the pelvic limbs [11, 12]. These connections coordinate flexion of one limb with extension of the other during the gait of the calf [11, 12]. Disruption of these connections leads to bilateral stimulation of the pelvic limbs which might also explain the simultaneous withdrawal reflex of the pelvic limbs at 2 weeks of age.

Over time, the calf showed deficits in the withdrawal and patellar reflexes of a seemingly worsening nature. The withdrawal reflex progressed from causing exaggerated flexion of both hind limbs, to being delayed and reduced and eventually absent in both pelvic limbs. The patellar reflex progressed from being normal to being delayed and reduced in both hind limbs. The delayed and reduced patellar reflex can be explained by the congenital anomaly in the spinal cord at the level of L4 [3]. Worsening of the clinical symptoms has most likely been caused by growth and therefore weight gain of the calf.

The bilateral muscle atrophy and stiffness in both pelvic limbs might be explained by the abnormal neural tone and function of the pelvic limbs. When muscles lack normal nervous tone, they remain underdeveloped, and as a consequence, the joints can be abnormal in their development and position which leads to fixated joints [13].

The calf also showed pollakiuria during the entire period of clinical examinations. This might have been caused by the congenital anomaly in the spinal cord at the level of L4. The hypogastric nerve, which originates from spinal segment L1-L4, stimulates the contraction of the internal urethral sphincter and inhibits the contraction of the detrusor muscle. Due to the anomaly in the spinal cord at the level of L4, the contraction of the M. detrusor might not have been inhibited adequately which might have resulted in the inability of storing large volumes of urine in the bladder. Additionally, the anomaly in the spinal cord at the level of L4 might have led to failure of contraction of the internal urethral sphincter, leading to passive urine loss. The combination of these two factors might have resulted in the clinical symptoms of active and passive pollakiuria in this calf.

### Cases of diplomyelia

Previously described cases in the literature were examined in comparison to the findings in the current case. In Hut et al. [1], the calf with diplomyelia showed symptoms of a general proprioceptive ataxia, paraparesis and an absent patellar reflex of the left pelvic limb. In Testoni et al. [2], the calf with diplomyelia was unable to stand and walk. Other cases of different types of myelodysplasia have also been diagnosed in calves. Calves with myelodysplasia have been described with varying symptoms like: sensory loss, non-progressive ataxia, ‘bunny hopping’, hypermetria, weakness in the hindlimbs and blindness [7]. Occurrence of myelodysplasia in the breeds Charolais and Hereford is thought to be higher compared to other breeds [7]. In Górriz-Martín et al. [14], three cases were described of calves with non-ambulatory paraparesis due to diplomyelia located in the lumbar vertebral column. One of these calves showed a congenital generalized low-frequency tremor. Moreover, a calf with ambulatory paresis was presented due to diastematomyelia, an anomaly that has a very similar development to diplomyelia during embryogenesis. In this article, pedigree analysis was performed on three cases. This analysis supported the hypothesis that the split spinal cord malformations are hereditary through a recessive mode of inheritance.

### Aetiology of diplomyelia

Congenital malformation of the spinal cord can be caused by genetic disorders, as mentioned above. Glia Cells Missing genes (GCM genes) have been thought to be involved in congenital malformation of the spinal cord. A study by Nait-Oumesmar et al. [15] showed that there might be a relationship between spina bifida, diastematomyelia and expression of GCM genes.

In-utero infection with certain pathogens can also possibly lead to various congenital malformations in the central nervous system of calves, such as: Schmallenberg virus (SBV), bovine virus diarrhea virus (BVDV), blue tongue virus (BTV), Akabane virus (AKAV), and Aino virus (AV) [16]. The calf, described in this case report, tested positive for Schmallenberg virus antibodies, however this does not necessarily imply that the virus caused the malformation of the spinal cord. A positive serology for Schmallenberg virus antibodies can be caused by passive (maternal) immunity or by active immunity (infection with the virus). If the calf has acquired the antibodies through active immunity, the symptoms of a Schmallenberg virus infection depend on the age at which the foetus has been exposed to the Schmallenberg virus. In addition, the Schmallenberg virus has been shown to result in anatomic malformations in the central nervous system, which is most often associated with a decrease in the amount of tissue [16, 17]. However, that was not the case in this calf. Thus, the cause of the focal diplomyelia at the level of L4 remains unknown.

## Supplementary information

**Additional file 1.** Gait abnormality of the calf at 8 weeks of age. Movie of the gait abnormality of the calf at 8 weeks of age.

## Data Availability

Not applicable.

## Abbreviations

AKAV: Akabane virus
AV: Aino virus
BHV-1: Bovine Herpes Virus-1
BTV: Blue tongue virus
BVDV: Bovine Viral Diarrhea Virus
GCM: Glia cells missing
L4: Fourth lumbar vertebra
L5: Fifth lumbar vertebra
L6: Sixth lumbar vertebra
MRI: Magnetic Resonance Imaging
SBV: Schmallenberg Virus
S1: First sacral vertebra
